# Remineralization effect of three different agents on initial caries and erosive lesions: a micro-computed tomography and scanning electron microscopy analysis

**DOI:** 10.1186/s12903-023-02805-6

**Published:** 2023-02-16

**Authors:** Sibel Akküç, Gülsüm Duruk, Ali Keleş

**Affiliations:** 1 Department of Pediatric Dentistry, Faculty of Dentistry, Inonu University, Malatya, Turkey; 2 Department of Endodontic, Faculty of Dentistry, Ondokuz Mayıs University, Samsun, Turkey

**Keywords:** Micro-CT, SEM, Erosion, Demineralization, Remineralization, Fluoride, Herbal, CPP-ACP, Toothpaste

## Abstract

**Background:**

This study aimed to investigate the remineralization efficiency of Sensodyne Promine containing Sodium flouride (NaF), GC Tooth Mousse containing CPP-ACP, and Agarta herbal toothpaste on initial caries and erosion using micro-computed tomography (CT) and scanning electron microscopy (SEM).

**Methods:**

Forty-five third-molar teeth for micro-CT were divided into three main groups after initial scans (*T*_1_) were completed. Artificial caries lesions were created with the demineralization cycle (group 1, n = 15) and artificial erosion lesions were created with orange juice (group 2, n = 15) and Cola (group 3, n = 15), and second scans (*T*_2_) were performed. The groups were divided into three subgroups within themselves. Sensodyne Promine toothpaste (subgroup 1a, 2a, 3a), GC Tooth Mousse topical cream (subgroup 1b, 2b, 3b), and Agarta herbal toothpaste (subgroup 1c, 2c, 3c) were applied using soft-tipped brushes for 2 min, twice per day for 15 days, and then a third scan (*T*_3_) was performed. Mineral density, surface area, and lesion volume and depth were calculated using micro-CT. Changes in the surface morphology of the teeth were examined using SEM in 13 samples representing each group, subgroup, and healthy enamel. In the analysis of the data obtained from the scans performed at three different times (*T*_1_, *T*_2_, *T*_3_), one-way analysis of variance (ANOVA) with the post-hoc Tukey test, repeated measures ANOVA with the post-hoc Bonferroni test, and paired sample *t*-test analyses were used.

**Results:**

All three agents caused a statistically significant increase in mineral density, and a decrease in surface area and lesion volume and depth (*p* < 0.05). There was no statistically significant difference between the groups in remineralization efficiency (*p* > 0.05). A statistically significant difference was found between the groups regarding the mineral density of the tissue that increased after remineralization (NaF > CPP-ACP > He; *p* < 0.05).

**Conclusion:**

The remineralization efficacy of herbal toothpaste as an alternative to NaF and CPP-ACP was found to be successful.

## Introduction

Enamel, which is the hardest tissue of the body, has a microporous structure due to the pores between the prisms [1]. This microporous structure of the enamel keeps the demineralization and remineralization balance dynamic by providing ion exchange between the oral environment, i.e., saliva, plaque, and the tooth surface [2]. When the intraoral environment drops below the critical pH (5.5), the demineralization/remineralization balance shifts in favor of demineralization as a result of the removal of calcium (Ca^2+^) and phosphate (PO_4_^3−^) ions from the tooth surface. In the process that follows, cavitation occurs on the enamel surface, but the enamel can be healed with non-invasive protective methods without cavitation, and the balance can be turned in favor of remineralization [3].

Nowadays, demineralization occurs in children as a result of increased dental caries due to high-carbohydrate dietary habits, and increased tooth erosion due to frequent consumption of fruit juices and carbonated beverages [4, 5]. It is known that sodium fluoride provides remineralization against demineralization resulting from initial caries, by forming fluorapatite together with calcium and phosphate ions [6]. There are many in-vitro studies examining the efficacy of remineralization agents against initial caries with different methods as an alternative to sodium fluoride [7–12]. In addition, the remineralization processes of initial caries and erosion lesions with different etiologies also differ [13]. Although fluoride products are frequently used, fluoride has a limited preventive effect against tooth erosion, because the effect is limited to demineralized enamel layers near the surface, the very low pH, and the absence of biofilm in erosion lesions [14, 15]. Studies are proving the effectiveness of remineralization agents against erosion alternatives to fluoride [16–19].

Advanced technological methods, determination of demineralization in subclinical stages, and evaluations of the effectiveness of enamel remineralization agents are currently provided [20–24]. Many studies have used scanning electron microscopy (SEM) as a qualitative method to assess the surface morphology of initial caries lesions [25] and erosion lesions [26]. In addition, non-destructive techniques allow long-term evaluation of the effects of remineralizing agents on enamel. Micro-computed tomography (micro-CT) is one of these methods used in many studies in dentistry. Micro-CT is a promising method for evaluating demineralization or remineralization, thanks to its reproducibility without damaging tissues, and the ability to obtain three-dimensional (3D) images and perform detailed analyses such as volume, depth, surface area, and mineral density [27–29].

In dentistry, which aims to increase the number of healthy teeth in the mouth, protective and preventive treatments have become widespread because modern diagnostic methods can detect the loss of hard tissue in the teeth while they are still at the mineral level. Oral and dental health is directly effective in the growth and development of children. It is expected that the incidence of dental diseases in children will decrease by starting preventive practices in the early period [30]. This study aimed to examine the remineralization efficiency of sodium fluoride (NaF)-containing Sensodyne Promine toothpaste, casein phosphopeptide amorphous calcium phosphate (CPP-ACP)-containing GC Tooth Mousse topical cream, and Agarta herbal toothpaste using micro-CT and SEM against the erosive lesions caused by acidic beverages frequently consumed by children, and artificial initial caries lesions. The null hypothesis of this study (*H*_0_) was that there would be no statistical difference between the remineralization efficiencies of the agents used for remineralization against both erosive and initial caries lesions.

## Methods

### Study design

**Fig. 1:**
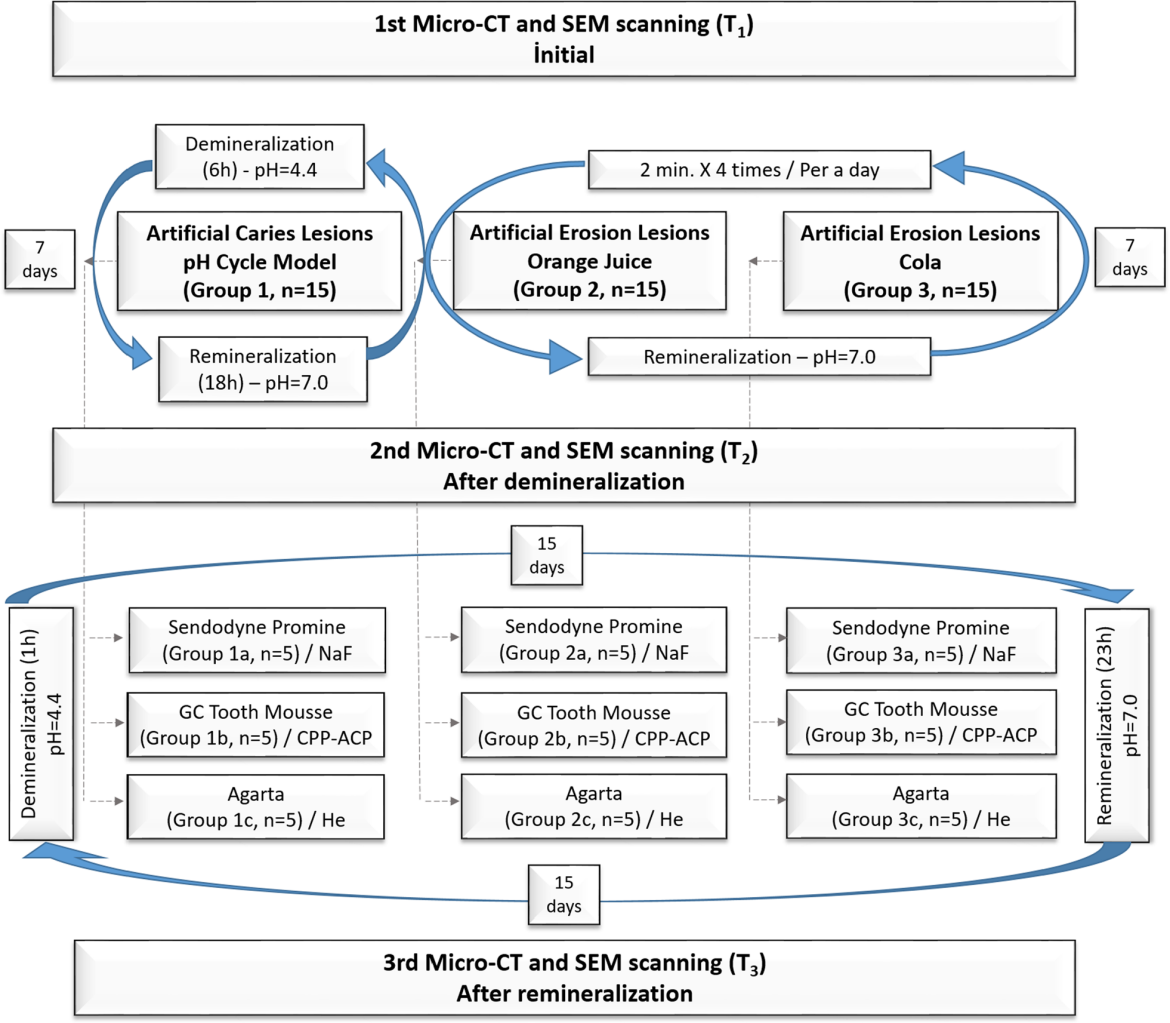
Flow chart of the experimental design of the study

Ethics committee approval for the study was obtained from Inonu University Scientific Research and Publication Ethics Committee (2021/1845). According to the power analysis performed regarding the relevant article [10], the minimum sample size was found as four in each subgroup, the type I error (alpha) amount was 0.05, the power (1-beta) of the experiment was 0.8, and the effect size was 2.6. In the study, the total sample size for micro-CT analysis was determined as 45, with five samples for each subgroup. The samples were divided into three main groups, “artificial caries lesions” (group 1), “artificial erosive lesions created by orange juice” (group 2), and “artificial erosive lesions created by Cola” (group 3). Each group had three subgroups, Sensodyne Promine toothpaste (subgroups 1a, 2a, 3a), GC Tooth Mouse topical cream (subgroups 1b, 2b, 3b), and Agarta toothpaste (subgroups 1c, 2c, 3c). Samples were randomly assigned to the groups (Fig. 1).

### Sample preparation

Forty-five third-molars were used for micro-CT analysis, and seven for SEM analysis. Fifty-two permanently impacted third molars with pre-planned extraction were collected. Decayed, fractured, cracked, hypocalcified, and hypomineralized teeth were excluded from the study. Tissue residues on the surface of the teeth were removed using a scaler (Hu-Friedy Mfg. Co., LLC, USA). The teeth extracted at most 1 month ago were stored in a saline solution containing 0.1% thymol at + 4 °C until the day of the experiment. For micro-CT analyses, the teeth were separated from their roots using separate discs below the enamel-cementum boundary and placed on acrylic blocks (Integra Self Cure Acrylic-Liquid, BGD, Turkey). Reference grooves were created on acrylic and the lingual surfaces of the teeth to standardize them in repetitive scans. Labels (3 × 3 mm) were attached to the buccal surfaces of the teeth and the remaining tooth surfaces were painted with acid-resistant nail varnish. Thus, a 9 mm^2^ experimental area was created. For SEM analyses, seven teeth were separated from their roots at the furcation border, and their crowns were divided into two at the level of the central fossa. Fourteen half teeth were obtained and 13 were used as samples. The teeth were placed on acrylic blocks with their buccal and lingual surfaces uppermost.

### Application of the experiment

The flow chart of the experiment is shown in Fig. 1. Following the initial scans (*T*_1_), demineralization lesions were created using an in vitro pH cycling model for 7 days, followed by a second scan (*T*_2_). To create an artificial caries lesion in group 1, the samples were exposed to the demineralization solution (pH = 4.4; 2.2 mM CaCl_2_, 2.2 mM NaH_2_PO_4_, 0.05 M CH_3_COOH, 1 M KOH) for 6 h and the remineralization solution (pH = 7.0; 1.5 mM CaCl_2_, 0.9 mM NaH_2_PO_4_, 0.15 M KCl, 1 M KOH) for 18 h [31]. To create erosion, the samples were exposed to 50 mL of Cappy Orange Juice (citric acid, pH = 3.34, TA = 5.01×10^−4^, The Coca-Cola Company®) in group 2, and 50 mL of Coca-Cola (phosphoric acid, pH = 2.24, TA = 5.75×10^−3^, The Coca-Cola Company®) in group 3, for 2 min, four times per day, and to the remineralization solution for the remainder of the day [32, 33]. For all groups, the solutions used were at room temperature.

The three agents used in the remineralization process are shown in Table 1. Two of the agents are used as children's toothpaste and the other is used as a remineralization agent in children. The teeth were brushed twice per day for 2 min, using a soft-tipped dental brush (Voco Comp.). This 4-min brushing time every 24 h was ignored. The samples were placed in a demineralization solution for 1 h per day to simulate the exposure of children to acids produced by cariogenic bacteria at meals; for the remaining 23 h, they were exposed to a remineralization solution to simulate saliva buffering. After providing remineralization for 15 days, micro-CT and SEM analyses were performed using images obtained from third scans (*T*_3_).

**Table 1:** Tooth agents used to provide remineralization

Type of remineralization agents	Tooth agents used in the experiment	Ingredients in toothpastes
Sodium Fluoride (NaF)	Sensodyne Promine (GlaxoSmithKline, USA)	Sorbitol, Aqua, Glycerin, Hydrated Silica, Polietilen Glikol (PEG-6), Cocomidoproply Betaine, Xanthan Gum, Aroma, Sodium Fluoride (1450 ppm 0.315%), Sodium Saccharin, Sucralose, Titanium Dipxide, Sodium Hydroxide, Limonene
Casein Phosphopeptide- Amorphous Calcium Phosphate (CPP-ACP)	GC Tooth Mousse (GC AMERICA INC, USA)	Water, glycerol, 10% CPP-ACP, D-sorbitol, CMC-Na,(GC Corporation, Japan) propylene glycol, silicon dioxide, titanium dioxide, xylitol, phosphoric acid, flavor, zinc oxide, sodium saccharin, ethyl p-hydroxybenzoatemagnesium oxide, guar gum, propyl, p-hydroxybenzoate, butyl p-hydroxybenzoate
Herbal (He)	Agarta (Güneyce LTD. TURKEY)	Sorbitol, Aqua, Glycerin, Silica, Lauryl Glucoside, Zinc Oxide, Aroma, Xanthan Gum, Mentha Piperita Oil, Saliva Officinalis Oil, Malaleuca Alternifolia, Leaf Oil, Potassium Sorbate

### Micro-CT analysis

In this study, 45 samples were scanned at three different times (*T*_1_, *T*_2_, *T*_3_) using the desktop SkyScan 1172 (Bruker-microCT, Kontich, Belgium) micro-CT system; a total of 135 scans were performed. The samples were placed in the same position thanks to the references in each scan and were scanned under standard conditions (80 kV, 124 mA, 0.5 mm Al-Cu filter, 11 MP camera, 0.4° rotation step, 200° rotation angle, 45–55 min). The images were converted from TIFF format to BMP format, restructured using the NRecon v. 1.7.4.2 software (Bruker-microCT) (75% correction, 7-unit ring artifact correction, and 3-unit smoothing), and made ready for analysis.

**Fig. 2:**
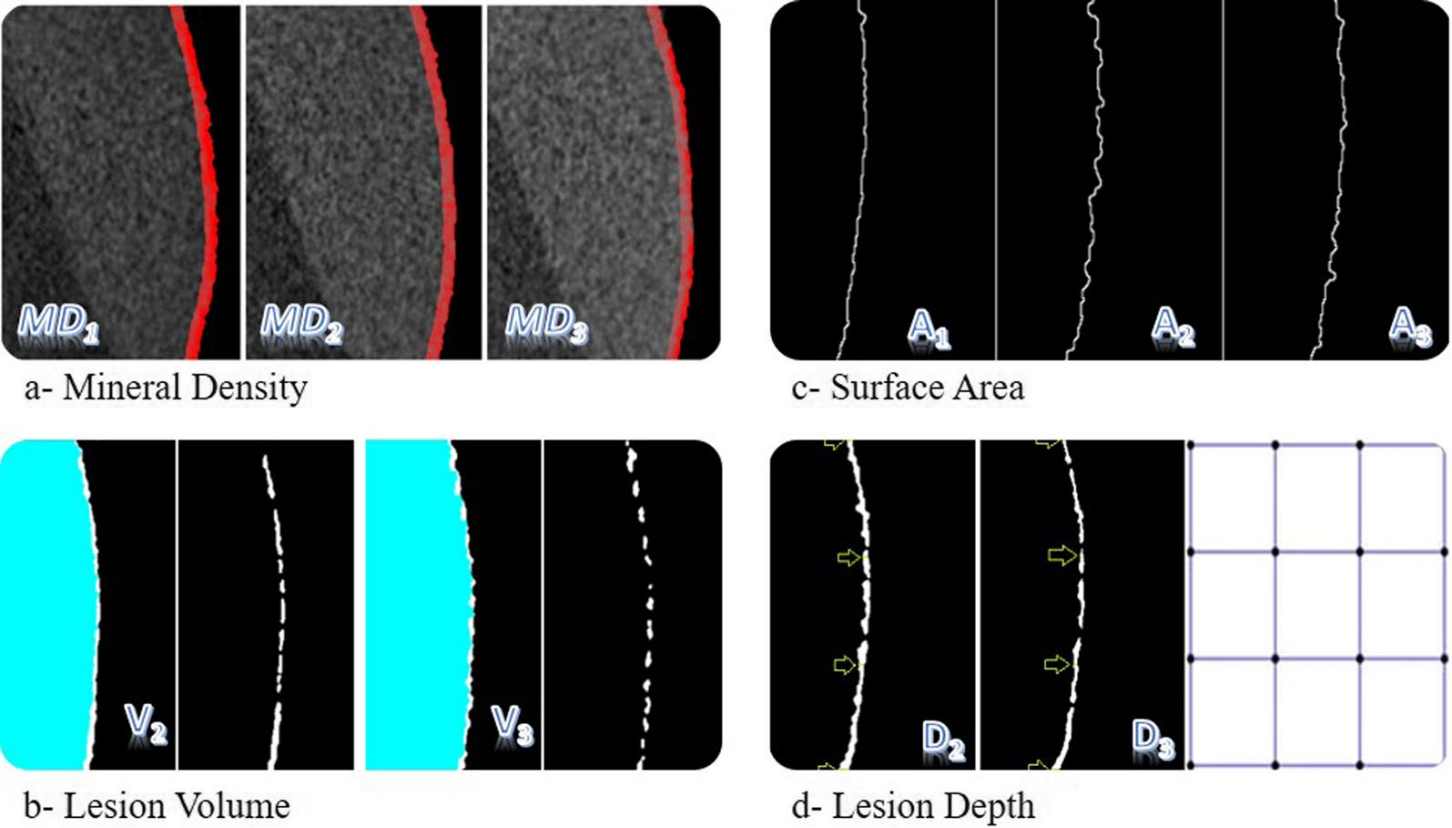
Micro-CT analysis stages

**Fig. 3:**
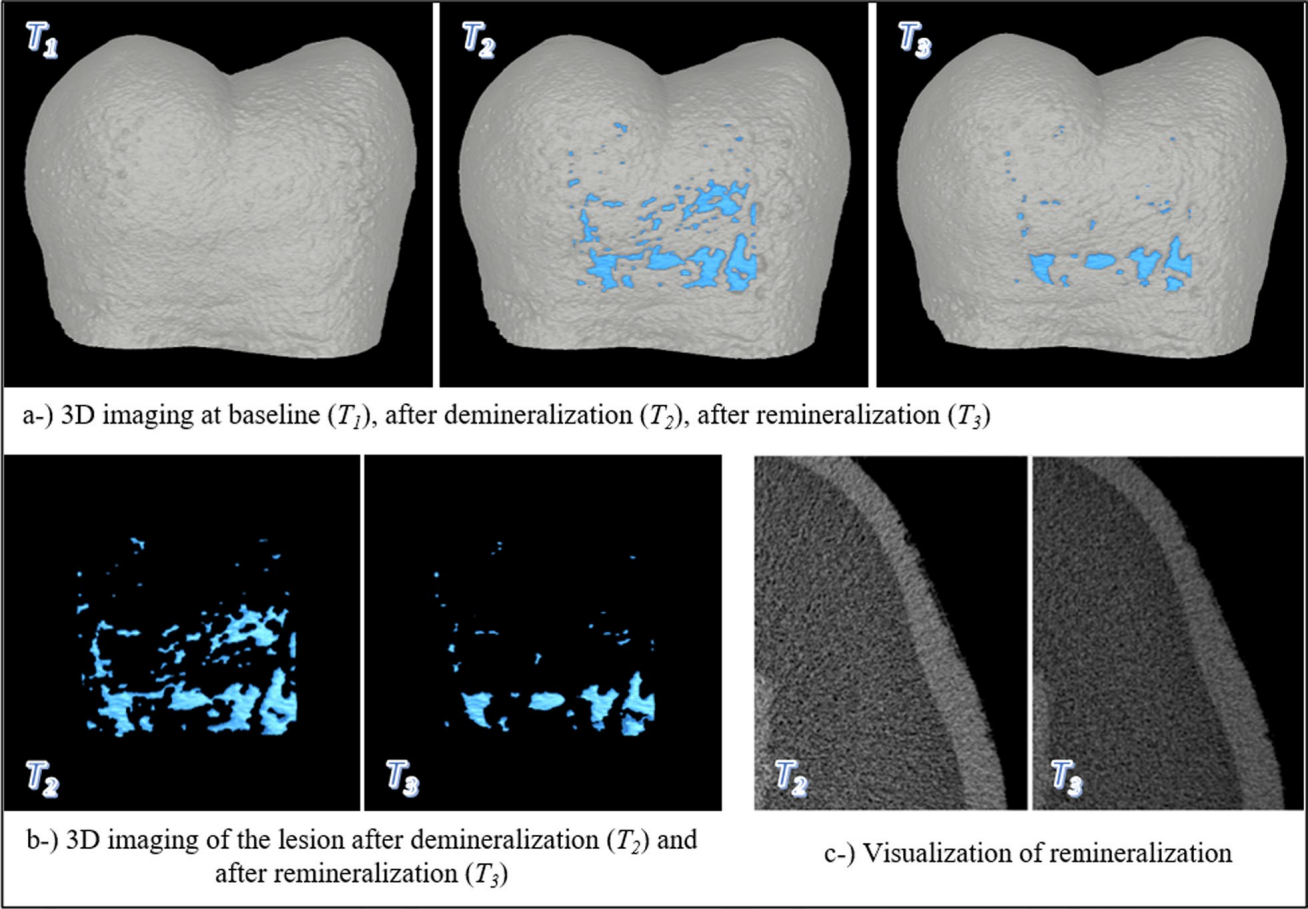
Creation of dimensional images

The images were positioned according to the reference grooves on the teeth (*T*_1_*-T*_2_, *T*_1_*-T*_3_, *T*_2_*-T*_3_) using the DataViewer v.1.5.6 software (Bruker-microCT), in the same way in three coronal, sagittal, and transversal axes in space, and new datasets were created by removing any unnecessary background. In the new dataset, a 2.5 × 2.5-mm working area was determined to be inside the experimental area using the CTAn v.1.18.8 software (Bruker-microCT). Mineral density calibration was performed with a previously scanned mineral reference phantom (Phantoms, Ratoc System Engineering, Tokyo, Japan) (MD = 0.25 and 0.75 g/cm^3^). Mineral density (*MD*) analysis was measured at two different depth intervals, (i) 100 μm from the outer margin of the enamel and (ii) 100 μm from the lesion base. Mineral density between 0–0.1 mm depth and 0.1–0.2 mm depth (*MD*_1_, *MD*_2_, *MD*_3_; g/cm^3^) was calculated (Fig. 2a). The volume of the lesion (*V*_2_, *V*_3_; mm^3^) was calculated by positioning the post-demineralization (*T*_1_*-T*_2_) and post-remineralization (*T*_1_*-T*_3_) images relative to the baseline (Fig. 2b). The surface area of the determined experiment area was calculated at three different times (*A*_1_, *A*_2_, *A*_3_; mm^2^) (Fig. 2c). The depth of the lesion was measured at 16 standard points (*D*_2_, *D*_3_; mm) from four points at equal distances from each other in the coronal and sagittal sections to represent the entire surface (Fig. 2d). When the images in T_2_ and T_3_ were matched in micro-CT scans, some tissue increase was found as a result of remineralization, this is shown in Fig. 3. The mineral density of this newly formed tissue was measured and calculated as ∆MD. 3D images were created using the CTVol 2.3.2.0 software (Bruker-microCT) for imaging from all the datasets obtained (Figs. 2, 3).

### SEM analysis

A total of 13 samples, healthy enamel (n = 1), four samples from groups 1, 1a, 1b, 1c, four samples from groups 2, 2a, 2b, 2c, and four samples from groups 3, 3a, 3b, 3c, were placed on the spray device after being fixed on the metal carrier. All surfaces were covered with gold–palladium (Au–Pd) and scanned in an SEM (LEO-EVO 40/Cambridge-England) operating at 20 kV filament voltage at 150 mA high vacuum. Images were taken from the experiment area on each sample at magnifications of × 2500, × 5000, and × 10,000.

### Statistical analysis

The IBM SPSS Statistics for Windows, V22 software (SPSS Inc., Chicago, IL, USA) was used for the statistical analyses. The data were first analyzed for normal distribution using the Shapiro*–*Wilk test. One-way analysis of variance (ANOVA) with the post-hoc Tukey test, repeated-measures ANOVA with the post-hoc Bonferroni test, and paired-sample *t*-test analyses were used to determine the remineralization efficiency of the agents in terms of the factors such as volume, depth, mineral density, and surface area at three different times (*T*_1_, *T*_2_, *T*_3_). *p* < 0.05 values were considered significant.

## Results

### Micro-CT results

The data of mineral density (Tables 2, vi6), volume (Table 3), surface area (Table 4), and lesion depth (Table 5) according to micro-CT analyses are presented in the relevant tables. For data in Tables 2 and 4, statistical double and triple comparisons were made. The p-value between *T*_1_ and *T*_2_ shows the effectiveness of demineralization, and the p-value between *T*_2_ and *T*_3_ shows the effectiveness of remineralization (Tables 2 and 4).

**Table 2:** Mineral densities (g/cm^3^) in the range of 0–0.1 mm and 0.1–0.2 mm; initial (*MD*_1_), after demineralization (*MD*_2_) and after remineralization (*MD*_3_)

Scanning time	*T*_1_	*T*_2_	*T*_3_	*T*_3_*-T*_2_		*T*_1_ *&T*_2_	*T*_2_ *&T*_3_
	*MD*_1_ Mean ± SD	*MD*_2_ Mean ± SD	*MD*_3_ Mean ± SD	Mean difference	**p	***p	***p
*0–0.1 mm*							
Group 1							
Subgroup 1a	2.329 ± 0.184^a^	1.765 ± 0.232^ab^	1.843 ± 0.155^b^	0.08 ± 0.10	0.019	0.023	0.144
Subgroup 1b	2.266 ± 0.090^a^	1.788 ± 0.145^b^	1.926 ± 0.053^b^	0.14 ± 0.13	0.001	0.008	0.083
Subgroup 1c	2.282 ± 0.165^a^	1.741 ± 0.172^b^	1.759 ± 0.171^b^	0.02 ± 0.02	< 0.001	0.002	0.065
*p	0.793	0.917	0.177	0.186			
Group 2							
Subgroup 2a	2.373 ± 0.085^a^	1.635 ± 0.207^b^	1.893 ± 0.266^b^	0.26 ± 0.19	< 0.001	0.001	0.039
Subgroup 2b	2.315 ± 0.070^a^	1.572 ± 0.251^b^	1.919 ± 0.172^b^	0.34 ± 0.30	< 0.001	0.001	0.063
Subgroup 2c	2.018 ± 0.209	1.633 ± 0.139	1.660 ± 0.139	0.03 ± 0.03	0.019	0.020	0.091
*p	0.777	0.877	0.144	0.078			
Group 3							
Subgroup 3a	2.016 ± 0.148^a^	1.590 ± 0.138^b^	1.713 ± 0.075^b^	0.12 ± 0.11	0.002	0.014	0.076
Subgroup 3b	2.070 ± 0.128^a^	1.685 ± 0.113^b^	1.757 ± 0.132^ab^	0.07 ± 0.08	0.003	0.010	0.124
Subgroup 3c	2.289 ± 0.181^a^	1.669 ± 0.157^b^	1.875 ± 0.107^ab^	0.21 ± 0.22	0.001	0.002	0.111
*p	0.104	0.539	0.082	0.409			
*0.1–0.2 mm*							
Group 1							
Subgroup 1a	2.478 ± 0.229	2.378 ± 0.199	2.414 ± 0.205	0.04 ± 0.03	0.003	0.020	0.031
Subgroup 1b	2.450 ± 0.125^a^	2.331 ± 0.125^b^	2.393 ± 0.113^a^	0.06 ± 0.03	0.001	0.008	0.015
Subgroup 1c	2.453 ± 0.148^a^	2.241 ± 0.145^b^	2.286 ± 0.119^c^	0.04 ± 0.04	0.001	0.010	0.060
*p	0.964	0.398	0.403	0.498			
Group 2							
Subgroup 2a	2.556 ± 0.109^a^	2.395 ± 0.079^b^	2.435 ± 0.091^c^	0.04 ± 0.02	< 0.001	0.002	0.013
Subgroup 2b	2.516 ± 0.042^a^	2.237 ± 0.084^b^	2.340 ± 0.108^a^	0.10 ± 0.04	< 0.001	0.003	0.004
Subgroup 2c	2.219 ± 0.192	1.980 ± 0.219	2.149 ± 0.169	0.17 ± 0.18	0.019	0.038	0.097
*p	0.881	0.233	0.490	0.188			
Group 3							
Subgroup 3a	2.235 ± 0.199	2.111 ± 0.119	2.167 ± 0.134	0.06 ± 0.05	0.020	0.050	0.075
Subgroup 3b	2.332 ± 0.400	2.077 ± 0.215	2,234 ± 0.321	0.16 ± 0.22	0.151	0.145	0.191
Subgroup 3c	2.546 ± 0.152	2.238 ± 0.147	2.411 ± 0.141	0.17 ± 0.18	0.009	0.024	0.102
*p	0.211	0.310	0.218	0.513			

*One One-Way ANOVA, Post-Hoc Tukey; **Repeated Measures ANOVA, Post-Hoc Bonferroni; ***Paired Sample *t*-Test

*p* < 0.05 was considered statistically significant

a-c: Different letters show statistically significant differences in the same row according to Repeated Measures ANOVA, Post-Hoc Bonferroni test

**Table 3:** Lesion volumes (mm^3^); after demineralization (*V*_2_) and after remineralization (*V*_3_)

Scanning time	*T*_1_*-T*_2_	*T*_1_*-T*_3_	Mean Difference	**p
	*V*_2_ Mean ± SD	*V*_3_ Mean ± SD	Mean ± SD	
Group 1				
Subgroup 1a	0.077 ± 0.004	0.036 ± 0.013	0.041 ± 0.013	0.002
Subgroup 1b	0.250 ± 0.037	0.173 ± 0.044	0.077 ± 0.046	0.020
Subgroup 1c	0.176 ± 0.050	0.120 ± 0.016	0.055 ± 0.043	0.045
*p	1.000	1.000		
Group 2				
Subgroup 2a	0.283 ± 0.031	0.183 ± 0.098	0.100 ± 0.087	0.062
Subgroup 2b	0.238 ± 0.039	0.203 ± 0.023	0.035 ± 0.023	0.028
Subgroup 2c	0.206 ± 0.026	0.166 ± 0.041	0.040 ± 0.033	0.057
*p	0.119	0.637		
Group 3				
Subgroup 3a	0.273 ± 0.015	0.242 ± 0.029	0.030 ± 0.021	0.037
Subgroup 3b	0.269 ± 0.014	0.260 ± 0.018	0.008 ± 0.005	0.035
Subgroup 3c	0.218 ± 0.016	0.163 ± 0.020	0.055 ± 0.023	0.006
*p	0.899	0.478		

*One Way ANOVA, Post-Hoc Tukey **Paired Sample *t*-test

*p* < 0.05 was considered statistically significant

**Table 4:** Surface areas (mm^2^); initial (*A*_1_), after demineralization (*A*_2_), and after remineralization (*A*_3_)

	*MD*_1_ Mean ± SD	*MD*_2_ Mean ± SD	*MD*_3_ Mean ± SD	Mean difference	**p	***p	***p
Group 1							
Subgroup 1a	9.980 ± 0.540^a^	11.705 ± 0.801^b^	10.390 ± 0.594^c^	1.32 ± 0.68	0.007	0.006	0.012
Subgroup 1b	10.328 ± 1.288^ab^	11.408 ± 0.957^a^	11.188 ± 0.876^b^	0.22 ± 0.12	0.052	0.038	0.015
Subgroup 1c	10.005 ± 0.629^a^	11.399 ± 0.532^b^	11.174 ± 0.617^b^	0.23 ± 0.19	0.001	0.012	0.059
*p	0.810	0.813	0.217	0.002			
Group 2							
Subgroup 2a	9.661 ± 0.636^a^	11.260 ± 0.339^b^	10.602 ± 0.817^ab^	0.66 ± 0.61	0.002	0.003	0.074
Subgroup 2b	10.051 ± 0.718^a^	11.258 ± 0.779^b^	10.775 ± 0.933^c^	0.48 ± 0.24	< 0.001	0.001	0.010
Subgroup 2c	9.833 ± 0.598^a^	11.264 ± 0.440^b^	11.103 ± 0.703^b^	0.16 ± 0.38	< 0.001	0.000	0.394
*p	0.625	1.000	0.613	0.230			
Group 3							
Subgroup 3a	10.548 ± 1.179^a^	10.884 ± 1.253^b^	10.779 ± 1.279^ab^	0.11 ± 0.07	0.002	0.010	0.032
Subgroup 3b	10.556 ± 1.377	11.024 ± 0.921	10.841 ± 1.033	0.18 ± 0.15	0.120	0.110	0.049
Subgroup 3c	9.615 ± 0.383^a^	10.070 ± 0.610^b^	9.973 ± 0.687^ab^	0.10 ± 0.30	0.037	0.015	0.508
*p	0.376	0.298	0.404	0.754			

*One-Way ANOVA, Post-Hoc Tukey; **Repeated Measures ANOVA, Post-Hoc Bonferroni; ***Paired Sample *t*-Test

*p* < 0.05 was considered statistically significant

a-c: Different letters indicate statistically significant differences in the same row according to Repeated Measures ANOVA, Post-Hoc Bonferroni test

**Table 5:** Lesion depth after demineralization (*D*_2_) and after remineralization (*D*_3_) (mm)

Scanning time	*T*_1_*-T*_2_	*T*_1_*-T*_3_	Mean Difference	**p
	*D*_2_ Mean ± SD	*D*_3_ Mean ± SD	Mean ± SD	
Group 1				
Subgroup 1a	0.015 ± 0.004	0.011 ± 0.004	0.003 ± 0.002	0.023
Subgroup 1b	0.033 ± 0.010	0.017 ± 0.009	0.016 ± 0.007	0.005
Subgroup 1c	0.025 ± 0.011	0.019 ± 0.009	0.005 ± 0.004	0.048
*p	0.375	0.347		
Group 2				
Subgroup 2a	0.045 ± 0.010	0.027 ± 0.010	0.018 ± 0.009	0.021
Subgroup 2b	0.031 ± 0.012	0.022 ± 0.009	0.009 ± 0.006	0.031
Subgroup 2c	0.033 ± 0.010	0.027 ± 0.009	0.005 ± 0.002	0.010
*p	0.170	0.553		
Group 3				
Subgroup 3a	0.034 ± 0.007	0.028 ± 0.004	0.005 ± 0.005	0.078
Subgroup 3b	0.030 ± 0.011	0.029 ± 0.012	0.001 ± 0.003	0.620
Subgroup 3c	0.025 ± 0.003	0.019 ± 0.002	0.005 ± 0.005	0.043
*p	0.288	0.133		

^*^One Way ANOVA, Post-Hoc Tukey **Paired Sample *t*-test

*p* < 0.05 was considered statistically significant

### Mineral density (*MD*) results

There was no statistical difference between the three agents used for remineralization in the change of mineral density both at 0–0.1 mm depth and 0.1–0.2 mm depth (*p* > 0.05). However, the mineral density decreased from *T*_1_ to *T*_2_ in all groups, and it increased as a result of brushing with all three agents from *T*_2_ to *T*_3_. There was a statistically significant difference within the group in all subgroups at 0–0.1 mm depth (*p* < 0.05). In the 0.1–0.2 mm depth, there was a statistically significant difference in all subgroups (*p* < 0.05), except subgroup 3b (*p* > 0.05). The decrease of mineral density in erosive lesions was higher than in initial caries lesions. The mineral density from *T*_1_ to *T*_2_ decreased in all three demineralization processes in the 0.1–0.2 mm depth, but not as much as in the 0–0.1 mm depth. The greatest density increase in the 0–0.1 mm depth (from 1.572 ± 0.251 to 1.919 ± 0.172 g/cm^3^) was in subgroup 2b (GC Tooth Mousse against orange juice). However, the least increase in mineral density from *T*_2_ to *T*_3_ at 0–0.1 mm depth was in Agarta toothpaste (subgroups 1c, 2c, 3c), and at 0.1–0.2 mm depth was in Sensodyne Promine. When the p values were examined according to the pairwise comparison between *T*_2_ and *T*_3_ (paired sample *t*-test) to evaluate the effectiveness of remineralization, statistically significant differences were found in subgroup 2a at 0–0.1 mm depth, in subgroup 1a, 1b, and 2b at 0.1–0.2 mm depth (Table 2).

### Volume (*V*) results

The mean (SD) lesion volume values measured after the demineralization (*V*_2_) and remineralization (*V*_3_) processes are presented in Table 3. There was no statistical difference in the change of lesion volume between the three agents used for remineralization (*p* > 0.05), but there was a statistically significant difference within the subgroups (*p* < 0.05), except for subgroups 2a and 2c. The lesion volume decreased after brushing with the three agents for remineralization in all groups. The greatest change in volume was in subgroup 2a (Sensodyne Promine against orange juice).

### Surface area (*A*) results

The initial surface area values (*A*_1_), after demineralization (*A*_2_), and after remineralization (*A*_3_) are presented in Table 4. The surface area increased as a result of demineralization in all groups. Although the surface area did not completely recover in all groups after brushing with the agents for remineralization, it decreased. After the remineralization process, there was a statistically significant decrease in the surface area in subgroups 1a, 1b, 2b, 3a, and 3b (*p* < 0.05). The most severe reduction in surface area (from 11.705 ± 0.801 mm^2^ to 10.390 ± 0.594 mm^2^) was observed in subgroup 1a (Sensodyne against initial caries), and the lowest reduction (from 10.070 ± 0.610 mm^2^ to 9.973 ± 0.687 mm^2^) was observed in subgroup 3c (Agarta against Cola) (Table 4). Sensodyne Promine toothpaste was found to be the most effective agent in reducing the surface area against artificial caries (*p* < 0.01).

### Depth (*D*) results

The mean (SD) lesion depht values measured after demineralization (*D*_2_) and remineralization (*D*_3_) are presented in Table 5. Although all three agents provided a decrease in the depth of all types of demineralization lesions, there was no statistically significant difference between the groups (*p* > 0.05). The decrease in depth observed within the subgroups except for subgroup 3a and subgroup 3b was found to be statistically significant (*p* < 0.05).

### ∆MD

A minimal enamel tissue increase was obtained after using agents for remineralization. When the mineral density of the newly deposited tissue was examined, a statistically significant difference was found between the three agents (group 1: *p* = 0.043, group 2: *p* = 0.001, and group 3: *p* = 0.003). Sensodyne provided the highest mineral density of all demineralization types, followed by GC Tooth Mousse and Agarta. When the accumulated tissue was evaluated in terms of the mineral density, the remineralization efficacy of the agents from high to low was Sensodyne Promine (NaF), GC Tooth Mousse (CPP-ACP), and Agarta (He), respectively (Table 6).

**Table 6:** Mineral density (*∆MD*) of new tissue formed from *T*_2_ to *T*_3_ (g/cm^3^)

Scanning time	*T*_2_*-T*_3_
	∆MD
Group 1	
Subgroup 1a	1.435 ± 0.171
Subgroup 1b	1.343 ± 0.210
Subgroup 1c	1,130 ± 0.122
*p	0.043*
Group 2	
Subgroup 2a	1.860 ± 0.216
Subgroup 2b	1.760 ± 0.181
Subgroup 2c	1.313 ± 0.125
*p	0.001*
Group 3	
Subgroup 3a	1.491 ± 0.069
Subgroup 3b	1.333 ± 0.092
Subgroup 3c	1.206 ± 0.134
*p	0.003*

*One Way ANOVA, Post-Hoc Tukey

*p* < 0.05 was considered statistically significant

The 3D imaging of the tooth at baseline, after demineralization and remineralization procedures, is presented in Fig. 3.

### SEM results

**Fig. 4:**
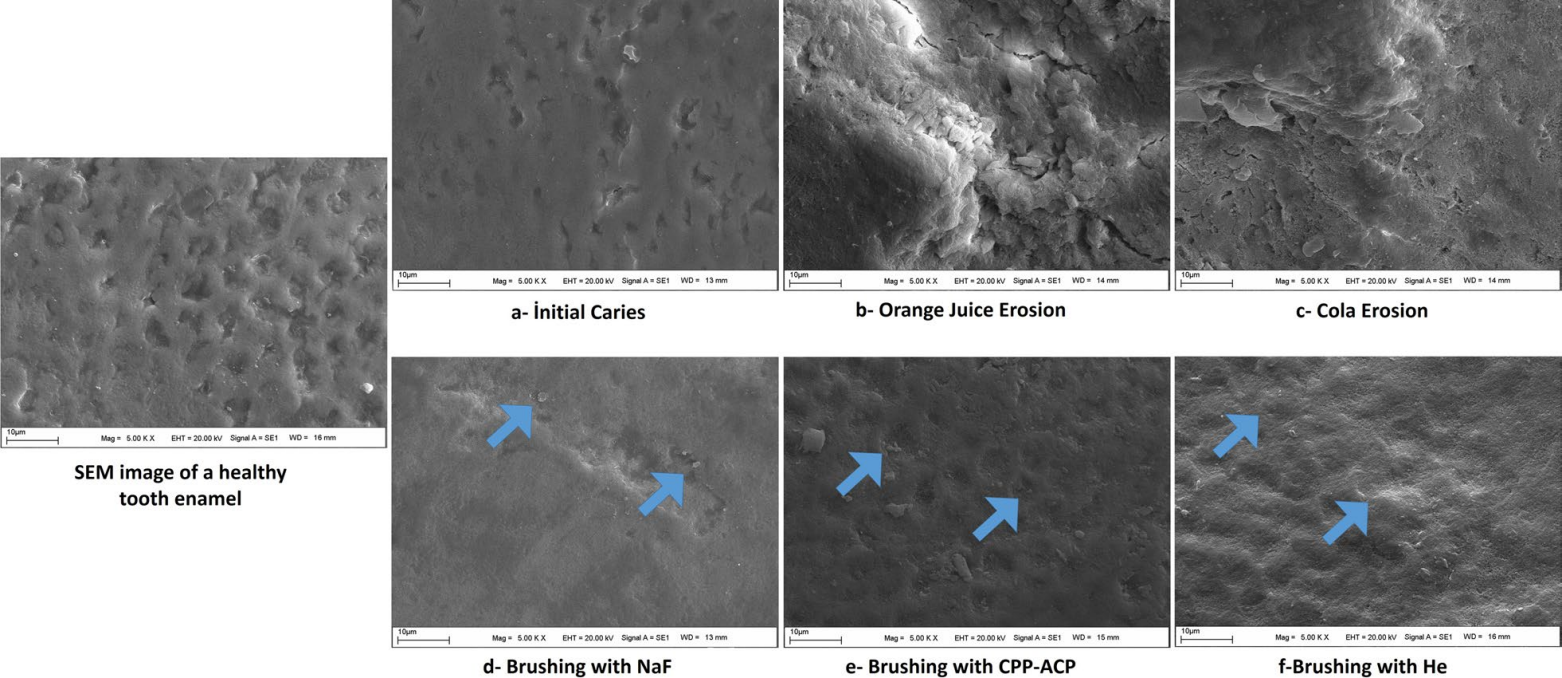
SEM analysis

In the presented study, a total of 39 SEM images were obtained from 13 samples at × 2500, × 5000, and × 10,000 magnifications. After demineralization (*T*_2_), it was observed that the keyhole-like appearance of the prisms in healthy enamel disappeared in artificial initial caries, the prism structure of the enamel was destroyed in orange juice erosion, and heterogeneous pits and intense porosity of the enamel surface integrity were observed in Cola erosion (Fig. 4). After remineralization (*T*_3_), against both caries and erosive lesions, (i) regional crystal deposition was obtained as a result of using Sensodyne Promine, (ii) the pores were covered as more heterogeneous and globular structures as a result of using GC Tooth Mousse, and (iii) the porous structure changed to a flatter surface as a result of using Agarta (Fig. 4).

## Discussion

Oral and dental health is directly effective in the growth and development of children. Inadequate oral and dental care can be prevented with primary preventive practices [30]. By starting the necessary primary preventive practices at an early age, the incidence of dental caries and dental erosion can be reduced [34]. Current scientific research in the field of preventive dentistry is about the prevention of demineralization, providing remineralization with non-invasive methods and diagnostic methods [35, 36]. In this study, the remineralization efficiency of three different remineralization agents for children against initial caries lesions and erosive lesions was evaluated simultaneously using micro-CT and SEM.

Scanning samples without causing damage, 3D imaging, performing sensitive, and detailed analyses are between the advantages of micro-CT. Its disadvantages can be listed as its being a time-consuming, costly, and information-intensive method. However, it is promising for future demineralization-remineralization studies.

Reynolds et al. [37] and Itthagarun et al. [29], who performed micro-CT scans in samples kept in demineralization solution for 4 days, observed lesions with a depth of 110–150 μm and 110–150 μm, respectively. Ten-Cate and Duijsters[38] and Vieira et al. [39] reported that a pH cycling regime created artificial caries 200 μm and 80–100 μm deep, respectively. In the present study, the depth of demineralization lesions ranged from 50 to 200 μm. The density decrease from *MD*_1_ to *MD*_2_ in the range of 0–0.1 mm and the range of 0.1–0.2 mm showed that the study was successful in creating artificial initial caries and erosive lesions. When the average mineral densities were examined between the groups, it was seen that the decrease in mineral density in erosive lesions was higher than in initial caries lesions (Table 2).

Barac et al. [40] reported that although Cola had a lower pH value, it had a higher corrosion potential immediately after exposure and decreased over time. However, orange juice caused more severe enamel erosion with long exposure. In our study, although orange juice had a higher pH and lower titratable acid value in the range of 0 to 0.1 mm depth, it caused more mineral loss than Cola. This is thought to be caused by a continuous long-term experiment cycle, as stated by Barac et al. Although the mineral loss in the range of 0.1–0.2 mm depth is also very low, the samples exposed to Cola caused more mineral loss than those exposed to orange juice at this depth. This shows that Cola can penetrate deeper.

In the present study, when the remineralization efficiency of the agents used was examined, it was observed that all three agents significantly increased the mineral density against demineralization and decreased the surface area, lesion volume, and depth of the lesion, but there was no statistically significant difference between the different kinds of toothpaste and topical cream (*p* > 0.05). However, the Agarta toothpaste provided the least increase in mineral density against initial caries and orange juice and Cola erosions. In addition, although the GC Tooth Mousse provided the greatest increase in mineral density against erosive lesions caused by orange juice, Sensodyne Promine was the most effective in reducing the volume, depth, and surface area of the lesion. However, the effectiveness of neither agent was statistically significant.

Hamba et al. [10] reported that experimental toothpaste containing TCP and fluoride most increased remineralization of artificial initial enamel carious lesions during pH cycles. In our study, when considering the remineralization efficiency (*T*_2_*&T*_3_) in subgroup 2a at 0–0.1 mm depth and subgroups 1a, 1b, and 2b at 0.1–0.2 mm depth were found to be statistically significant. This means that Sensodyne Promine and GC-Tooth Mousse cream are effective in increasing mineral density, but Agarta does not appear to be effective in remineralization. Our results are in agreement with Hamba et al.'s study [10]. In another study by Hamba et al. [11] the researchers applied CPP-ACP and CPP-ACFP to bovine teeth and reported that according to micro-CT analysis, the fluoride-containing group provided less mineral loss than the CPP-ACP group (*p* < 0.05). In our study, no statistically significant difference was found between Sensodyne Promine and GC-Tooth Mousse. On the other hand, GC-Tooth Mousse provided more effective remineralization than Sensodyne Promine against the erosion caused by orange juice. This may be due to the difference in the study designs regarding demineralization patterns, as well as the differences between human and bovine teeth. However, Ustun et al. [16] showed that CPP-ACFP had a superior effect in preventing enamel loss due to tooth erosion in primary teeth. Devadiga et al. [18] found that agents containing CPP-ACP and β-TCP increased hardness of erosive tooth surface. Findings of our study, which agreement with the literature [13–15], it is stated that GC-ToothMousse cream may be more effective against Sensodyne Promine and Agarta in erosion lesions due to its very low pH. It is thought that our study will be a reference on the micro-CT studies on the remineralization effect of CPP-ACP-containing creams on demineralization lesions caused by erosion.

Miyahira et al. [41] used tooth agents with fluoride, CPP-ACP, and CPP-ACP + fluoride in their study. Fluoride and CPP-ACP + fluoride exhibited similar results, and CPP-ACP increased the enamel mineral density and decreased the depths of lesions. Farooq et al. [42] brushed teeth with fluoride, bioactive glass, and distilled water in their in-vitro study, and revealed that the enamel volume decreased after demineralization and increased after remineralization in all groups according to micro-CT analysis. Similarly, in our study, Sensodyne Promine provided the greatest volume increase. In a micro-CT study, Nakata et al. [43] stated that as a result of demineralization, the surface area increased and showed an indented area, and flatter surfaces were observed as a result of remineralization. In our study, the surface became flatter and the area decreased after remineralization, similar to Nakata et al.'s study. Although there was no statistical difference, Agarta provided the least decrease in the lesion area, and the maximum decrease was in the Sensodyne promine against the initial caries group. In many studies, the effectiveness of fluoride and CPP-ACP have been proven [6, 13]. Nowadays, many parents tend to prefer herbal agents because of their chemical content. Therefore, parents may turn to GC Toothmousse cream containing casein phosphopeptide for their children, instead of opting for less effective herbal pastes versus fluoride-containing toothpastes.

When the mineral density of accumulated tissue was examined, a statistically significant difference was found between the groups. In all three groups in which demineralization was achieved in different ways, Sensodyne Promine provided the highest concentration of mineral density in the accumulated tissue, followed by the GC Tooth Mousse. Agarta provided the least density in the accumulated tissue. As a result of this study, although there was no statistically significant difference in remineralization efficiency between all three agents, more studies are needed on the properties of herbal toothpaste such as antibacterial efficacy, cytotoxic biocompatibility, and remineralization efficiency.

SEM is a good method for the qualitative measurement of surface morphology [44]. Although it is a disadvantage that the samples cannot be reused, being able to clearly examine the morphologic structure is among its advantages [45]. As in the study of Huang et al. [46], we observed non-homogeneous irregularities in the enamel prisms in the SEM images of initial caries formed by the pH cycle. Lussi et al. [47] reported that, as a result of abrasion with orange juice or citric acid, there was a loss on the surface and destruction in the form of 'etching.' Poggio et al. [48] stated that, after the samples were exposed to Cola for 2 min four times per day, the loss of material from the surface was remarkable and the SEM prism formed a honeycomb-like structure, similar to the present study. Both studies [47, 48] are similar to our study. In the present study, after brushing with NaF-containing toothpaste against demineralization, heterogeneous remineralized enamel was obtained on the surface characterized by crystal accumulation, and accumulations in the form of scattered granular deposits were obtained after brushing with CPP-ACP paste. Similar to our study, in the study by Altan et al. [49], SEM revealed a more homogeneous image on the remineralization surface formed with NaF-containing toothpaste, whereas CPP-ACP applied surfaces were more heterogeneous and some pits were not filled with minerals.

The limitations of this study were as follows: (i) the short duration of the experiment, (ii) the inability to fully reflect the in-vivo conditions, (iii) the inability to include primary teeth through the use of agents for children, (iv) errors that could occur due to positioning of the samples during micro-CT analysis, and (v) the need of standardization for artificial erosion and artificial caries lesion formation procedures. However, comparing the efficacy of three different agents for remineralization against initial caries and erosive lesions simultaneously using micro-CT and SEM, providing a reference for future micro-CT studies about demineralization-remineralization in terms of the method of study, and obtaining detailed information on remineralization with 3D comprehensive analyses were the strengths of the study.

Although there was no statistically significant difference between the groups with NaF, CPP-ACP, and herbal toothpaste agents against initial caries, and Cola and orange juice erosion, all agents provide an increase in mineral density and a decrease in lesion volume, depth, and surface area. Regular tooth brushing, which is among the primary steps of preventive medicine, can heal initial caries and erosive lesions. The remineralization effect of fluoride and CPP-ACP was supported by this study. The remineralization efficiency of agents such as herbal toothpaste can be supported by more studies and offered as an alternative in the future. More studies evaluating demineralization and remineralization using micro-CT are needed to shed light on many different areas in dentistry.

## Data Availability

The datasets generated and/or analysed during the current study are available from the corresponding author on reasonable request.

## Abbreviations

Ca^2+^: Calcium
CPP-ACP: Casein Phosphopeptide Amorphous Calcium Phosphate
Micro-CT: Micro-Computed Tomography
PO_4_^3^^−^: Phosphate
SEM: Scanning Electron Microscopy
NaF: Sodium Fluoride
